# Features of complementary and alternative medicine use by patients with coronary artery disease in Beijing: a cross-sectional study

**DOI:** 10.1186/1472-6882-13-287

**Published:** 2013-10-28

**Authors:** Fu-Yong Chu, Xue Yan, Ze Zhang, Xing-Jiang Xiong, Jie Wang, Hong-Xu Liu

**Affiliations:** 1 Department of Cardiology, Beijing Hospital of Traditional Chinese Medicine, Capital Medical University, Beijing, China; 2 Department of Psychology and Sleep Medicine, Guang An Men Hospital, China Academy of Chinese Medical Sciences, Beijing, China; 3 Department of Gerontology, Affiliated Hospital of Liaoning University of Traditional Chinese Medicine, Shenyang, Liaoning, China; 4 Department of Cardiology, Guang An Men Hospital, China Academy of Chinese Medical Sciences, Beijing, China

**Keywords:** CAM use, CAD, Cross sectional study, Patent herbal medicine

## Abstract

**Background:**

Complementary and alternative medicine (CAM) is commonly used in China for the management of coronary artery disease (CAD). However, few studies have been conducted to investigate the prevalence, perceived effectiveness, types, and reasons of CAM use in patients diagnosed with CAD.

**Methods:**

A cross-sectional study design was adopted. Questionnaires were distributed at the outpatient cardiac clinics of four tertiary-level teaching general hospitals in Beijing. Quantitative data were analyzed using Student’s *t*-test. Categorical data were analyzed using chi-square test. Logistic regression was employed to explore factors associated with the use of CAM as well as CAM use features in Chinese medicine (CM) hospitals when significant differences were found upon comparisons.

**Results:**

From May to July, 2009, a total of 600 questionnaires were distributed, and 546 patients with a diagnosis of CAD responded with valid values and were included in the present study. CAM was used by 69.1% of the patients with CAD; the majority (75.9%) of these CAM users believes that CAM is effective. “Few side effects” (49.6%) was the main reason of CAM use; whereas “doubt of effect” (61.5%) was the main reason for non-use. Patent herbal medicine (90.7%) was the most commonly used CAM type. Compared with non-CAM users, CAM users tended to be older (p < 0.01), have a longer disease duration (p = 0.02) and better current health status. In addition, CAM users had significant lower odds for emergency admission and hospitalization within the past one year. Patients with CAD from CM and WM hospitals differ in CAM use frequency, types, perceived effectiveness, as well as reasons for CAM use or non-CAM use.

**Conclusion:**

The present study suggested a group of significant factors which could influence the use of CAM in patients with CAD. CAM use patterns differ in patients from CM and WM hospitals.

## Background

Coronary artery disease (CAD) is the inflammatory result of the accumulation of atheromatous plaques within the walls of the coronary arteries which supply the heart with oxygen and nutrients [1]. Manifestations of CAD may include asymptomatic CAD, stable angina, unstable angina, acute myocardial infarction (MI), and sudden death. With the transition of people’s life style and prevalence of westernized diet, coronary artery disease (CAD) has become one of the most prevalent health problems worldwide [2]. CAD is the leading cause of death in the United States and the second leading cause of cardiovascular death in China [3,4]. In both countries which have the world’s largest economies, the prevalence and impact of CAD are expected to grow [3-5]. Management of CAD typically consists of smoking cessation, blood pressure control, lipid management, physical activity, weight management, and treatment of concomitant disorders [2]. However, even with the most up-to-date management including interventional or surgical techniques which have largely decreased the mortality of CAD, quality of life of these patients needs to be further addressed [5-7]. In addition, the direct care and significant mortality, disability, and loss of productivity of the disease impose enormous economic burden on families, societies, and the public health system of countries [8,9].

Complementary and alternative medicine (CAM) which is defined as health care practices or interventions which are not taught in conventional biomedical schools is commonly used by the general population all over the worlds [10]. Based on epidemiological studies, an increasing trend in CAM use exists in the United States [10], England [11], Canada [12-14], Australia [15], Israel [16], and South Africa [17]. Recent studies also confirmed the use of CAM in patients with CAD. Decker et al. [18] reported that 19% patients with acute coronary syndrome used CAM, and higher proportions of patients who used CAM were non-Caucasian, uninsured, economically burdened, and had depression. Prasad et al. [19] reported that 75.4% patients with cardiovascular disease used dietary supplements, 31.5% chiropractic therapy, 23.9% mind-body therapies, and 19.2% massage; however, only 14.4% had discussed the use of CAM treatments with their physicians. Grant et al. [20] summarized 27 studies regarding CAM use by patients with cardiovascular disease and found that CAM use in cardiac patients ranged from 4% - 61% with biologically-based therapies usage ranging from 22% to 68% and herbal medicines ranging between 2% and 46%. The heterogeneity of the results in those studies indicates that CAM use in patients varies in different countries, cities, ethnicities, specific CAM modalities, and patients’ economic status.

CAM therapies are commonly categorized into the following categories: whole medical systems, mind-body medicine, biologically based practices, manipulative and body-based therapies and energy medicine [21]. Traditional Chinese Medicine (TCM) which includes herbal medicine, acupuncture, qigong, and tai chi originated from China. Components of TCM may fit into one or several of CAM categories, for example, qigong may be regarded as an energy medical therapy, and Chinese herbal medicine may be perceived as the whole medical systems. As a requirement by Chinese government policies, acupuncture and herbal medicine are offered in the Departments of Acupuncture and/or Chinese Medicine (CM) inside almost every western medicine (WM) hospitals. Meanwhile, a large number of CM hospitals where almost all patients receive CM therapy exist in China. TCM has been used in China for the management of CAD for more than two thousand years. However, few studies have been conducted to investigate the prevalence, perceived effectiveness, types, and reasons of CAM use in patients diagnosed with CAD in China. In addition, to our best knowledge, no studies have been performed to explore the differences of CAM use between patients visiting CM and WM hospitals. Therefore, the aims of the present study were to: (1) determine the prevalence, perceived effectiveness, types, and reasons of CAM use in patients diagnosed with CAD in Beijing; (2) investigate the possible differences between CAM users and non-CAM users; (3) explore possible different CAM use features between CM and WM hospitals.

## Methods

### Study design and setting

After approval from the Human Research Committees at Guang An Men Hospital, An Zhen Hospital, Xi Yuan Hospital, and Xuan Wu Hospital, the present cross-sectional study was performed at the outpatient cardiac clinics of these four tertiary-level teaching general hospitals in Beijing, north China. The method of verbal informed consent only was approved by the hospital ethics committees. Although the Chinese population is exposed to the same Chinese culture, the majority of patients may be more likely to go to CM hospitals for CAM treatments. As the goal of the study is to capture the characteristics of CAM use in patients with CAD and compare the possible differences in CAM use between CM and WM hospitals, surveys were distributed simultaneously at two out of five tertiary-level CM hospitals and two out of 30 tertiary-level WM hospitals in Beijing. The two CM hospitals of Guang An Men Hospital and Xi Yuan Hospital are well-known by local Chinese for their CM treatment of CAD; whereas the two WM hospital of An Zhen Hospital and Xuan Wu Hospital are well-known by local Chinese for their WM treatment of CAD. At the outpatient cardiac clinic in each of the four hospitals, 150 CAD consecutive patients were approached and a verbal informed consent was gained from patients prior to participation. Patients were then instructed to complete the questionnaire at the clinic. Patients who filled the questionnaire before and patients who were illiterate were excluded at the site of survey. In addition, patients who used CAM for other purposes besides the management of CAD were also excluded.

### Diagnostic criteria of CAD

In the present study, CAD was defined as stable angina (SA), unstable angina (UA) and myocardial infarction (MI, including non-ST-elevation MI, ST-elevation MI, and old MI). The diagnostic criteria were based on the recommendations of the latest guidelines of American College of Cardiology and American Heart Association (ACC/AHA) [22-24]. All the patients were over 18 years old without any language or neurological disorders that may prevent them from voluntary participation in the study.

### Contents of the questionnaire

The questionnaire composed of two parts with 21 questions in total. Part One recorded the following three domains: (1) demographic characteristics of the patients (gender, age, place of residence, education level, and monthly household income); (2) CAD-related information (CAD types, duration, concomitant disorders, emergency admission or hospitalization within the past one year), and (3) CAM use characteristics (types, frequency, perceived effectiveness, reasons for use or non-use). Part Two recorded patients’ current health status via the Chinese version of Seattle Angina Questionnaire (SAQ), which had a good reliability and validity in Chinese patients [25]. Translation of Questions in Part One of the questionnaire includes:

1. What is the pattern (type) of your CAD? (done by the researchers as per patient reports and acquisition of patients’ medical history when necessary)

2. How long have you had CAD?

3. Do you have any concomitant disorders? If yes, what are they?

4. Have you ever had any emergency admission or hospitalization due to CAD within the past one year?

5. Have you ever used any types of CAM (such as patent herbal medicine, nutrient supplements, exercise, herbal decoction, acupuncture, and others) to treat your CAD? If yes, what are they?

6. How often did you use CAM to treat your CAD, continuous use, intermittent use, or used once?

7. After the treatment of CAM for CAD, do you think it is effective? Yes or No

8. Why do you take CAM to treat your CAD? (done by CAM users) Fewer side effects; effective and mild action; treating root cause; economic; health preserving effect; without invasive examination; others (please mark the one/s that best describe your condition).

9. Why don’t you take CAM to treat your CAD? (done by non-CAM users) Terrible taste; slower effectual procedure; afraid of fake CAM products; doubt of effect; inconvenient; afraid of acupuncture; others (please mark the one/s that best describe your condition).

10. Which types of CAM (e.g. patent herbal medicine, nutrient supplements, exercise, herbal decoction, acupuncture, and others) do you prefer, if you plan to use it in the future for the management of CAD? (done by non-CAM users) Note: Exercise refers to walking, jogging, or other physical activities longer than half an hour daily. Others refer to chiropractor therapy, yoga, massage, meditation, etc.

### Statistical analysis

Statistical analysis was performed with SPSS 13.0 software (SPSS Inc., Chicago, IL, USA) for windows. Quantitative data were expressed with mean ± standard deviation (SD) and were analyzed using Student’s *t*-test. Categorical data were described and analyzed using chi-square test. Logistic regression was employed to explore factors associated with the use of CAM as well as CAM use features in Chinese medicine (CM) hospitals when significant differences were found upon comparisons. A two tailed *P* < 0.05 was considered statistically significant. Demographic characteristics were described with actual number and percentages.

## Results

### Demographic characteristics of the patients

From May to July, 2009, a total of 600 questionnaires were distributed at the outpatient cardiac clinics of the four hospitals, and 546 patients with a diagnosis of CAD responded with valid values and were included in the present study. Characteristics of the included participants are detailed in Table 1. Fifty-four patients returned questionnaires which had no valid information or were non-legible and thus were excluded. In the sampled population, 308 (56.4%) patients were male, 238 (43.6%) patients were female; the age was 61.3 ± 9.2 years and the disease duration was 9.6 ± 6.3 years. Regarding specific diagnosis of CAD, 33.9% were diagnosed with SA, 61.2% were diagnosed with UA, and 4.9% were diagnosed with MI. The majority of the patients were urban citizens (74.2%). A greater portion of patients reported a monthly household income higher than 4,000 RMB (54.5%, one USD equaled 7 RMB at that time) and ≥ 2 concomitant diseases (55.9%), and had less than college education (76.2%).

**Table 1:** Demographic characteristics of the patients (n = 546)

**Demographic characteristics**	**N**
Gender (%)	
Male	308 (56.4)
Female	238 (43.6)
Age (years)	61.3 ± 9.2
CAD type (%)	
SA	185 (33.9)
UA	334 (61.2)
MI	27 (4.9)
Places of residence (%)	
Urban	405 (74.2)
Rural	141 (25.8)
MHI (%)^a^	
< 4000 RMB	248 (45.5)
≥ 4000 RMB	298 (54.5)
Education level (%)	
LC	416 (76.2)
CP	130 (23.8)
Disease duration (years)	9.6 ± 6.3
Numbers of concomitant diseases (%)	
≤ 2	241 (44.1)
> 2	305 (55.9)
Emergency admission (%)	
Yes	220 (40.3)
No	326 (59.7)
Hospitalization (%)	
Yes	307 (56.2)
No	239 (44.8)

CAD, Coronary artery disease; SA, Stable angina; UA, Unstable angina; MI, Myocardial infarction; MHI, Monthly household income; PS, Less than college education; CP, College and postgraduate; CAM, Complementary and alternative medicine.

^a^One US dollar equaled 7 RMB at that time.

### Comparisons between CAM use and non-CAM use groups

Based on whether the patient used CAM for the management of CAD or not, 546 CAD patients were divided into two groups: CAM use group (n = 377, 69%) and non-CAM use group (n = 169, 31%). No significant differences were found between CAM use and non-CAM use groups in terms of gender, places of residence, education level, and monthly household income, number of concomitant diseases, treatment satisfaction, and disease perception. Compared with the non-CAM use group, patients in the CAM use group tended to be older (*P <* 0.01), have a longer disease duration (*P* = 0.02), and better current health status as evaluated by SAQ (physical limitation: *P* = 0.01; angina stability: *P* = 0.03; angina frequency: *P* = 0.01). During the past one year prior to study participation, 138/377 (36.6%) participants in the CAM use group as compared to 82/169 (48.5%) participants in the non-CAM use group experienced emergency admission (p < 0.01); 201/377 (53.3%) participants in the CAM use group as compared to 106/169 (62.7%) participants in the non-CAM use group were hospitalized at least once (p = 0.04). Compared with non-CAM users, CAM users had significant lower odds for emergency admission (OR = 0.61, 95% CI = 0.42 - 0.89, *P* < 0.01) and hospitalization (OR = 0.68, 95% CI = 0.47 - 0.98, P = 0.04) within the past one year (see Table 2).

**Table 2:** Comparison between CAM use and non-CAM use groups

**Characteristics**	**CAM use (n = 377)**	**Non-CAM use (n = 169)**	**P**
Gender (male / female)	211 / 166	97 / 72	0.75
Age (years)	62.3 ± 10.2	59.5 ± 8.4	< 0.01
Disease duration (years)		8.6 ± 5.9	0.02
Places of residence (urban/rural)	10.2 ± 7.6	123 / 46	0.62
MHI^a^	282 / 95	83 / 86	0.25
(< 4000 RMB / ≥ 4000 RMB)	165 / 212		
Education level		127 / 42	0.70
(LC / CP)	289 / 88		
Number of concomitant disease		80 / 89	0.31
(≤ 2 / > 2)	161 / 216		
Emergency admission		82 / 87	< 0.01
(yes / no)	138 / 239		(OR = 0.61, 95% Cl = 0.42 - 0.89)
Hospitalization		106 / 63	
(yes / no)	201 / 176		0.04
CAD-type			(OR = 0.68, 95% Cl = 0.47 - 0.98)
SA		58	
UA	127	104	
MI	230	7	
SAQ-physical limitation	20	51.8 ± 9.9	0.57
SAQ-angina stability	59.4 ± 10.8	38.9 ± 13.5	
SAQ-angina frequency		54.5 ± 13.2	0.01
SAQ-treatment satisfaction	45.8 ± 14.2	65.8 ± 9.6	0.03
SAQ-disease perception	63.2 ± 14.7	47.6 ± 11.7	0.01
	67.4 ± 11.1		0.12
			0.36
	48.5 ± 12.6		

CAM, Complementary and alternative medicine; MHI, Monthly household income; LC, Less than college education; CP, College and postgraduate; SAQ, Seattle Angina Questionnaire.

^a^One US dollar equaled 7 RMB at that time.

### Prevalence, perceived effectiveness, reasons, and types of CAM in CAD Management

Information of prevalence, perceived effectiveness, reasons, and types of CAM in CAD management is provided in Table 3. In the present study, 377 (69.1%) patients had used at least one CAM therapy in treating CAD. Among these 377 patients, 132 patients used it continuously; 286 patients considered CAM effective after CAM use. Patent herbal medicine (90.7%) was the most commonly used type. Patent herbal medicine refers to extracted condensed pills or solutions from herbal medicine. The main reason of CAM use was “few side effects” (49.6%); whereas “doubt of effect” (61.5%) was the main reason for non-use. The majority of non-CAM users preferred patent herbal medicine (81.1%) and nutrient supplements (66.3%) as the future options if they would use CAM to treat CAD.

**Table 3:** CAM use information and comparisons between CM and WM hospitals

**Characteristics**	**N**	**CM hospital**	**WM hospital**	**P**
Patients analyzed	546	282	264	
CAM users (%)	377 (69.0)	257 (91.1)	120 (45.5)	<0.01
Frequency				
Continuous / intermittent or once	132 / 245	113 / 144	19 / 101	<0.01 (OR = 4.17, 95% CI = 2.41 - 7.22)
Patterns (%)				
Chinese patent prescription	342 (90.7)	237 (69.3)	105 (30.7)	0.14
Nutrient supplements	204 (54.1)	136 (66.7)	68(33.3)	0.50
Exercise	129 (34.2)	81 (62.8)	48 (37.2)	0.11
Herbal decoction	280 (74.3)	224 (80.0)	56 (20.0)	<0.01
Acupuncture	52 (13.8)	42 (80.8)	10 (19.2)	0.04
Others	18 (4.8)	13 (72.2)	5 (27.8)	0.71
Perceived effectiveness				
Effective / ineffective	286 / 91	217 / 40	69 / 51	<0.01 (OR = 4.01, 95% CI = 2.45 - 6.58)
Reasons for CAM use (n = 377)				
Fewer side effects	187 (49.6)	135 (72.2)	52 (27.8)	0.10
Effective and mild action	155 (41.1)	132 (85.2)	23 (14.8)	<0.01
Treating root cause	106 (28.1)	97 (91.5)	9 (8.5)	<0.01
Economic	79 (21.0)	58 (73.4)	21 (26.6)	0.26
Health preserving effect	37 (9.8)	27 (73.0)	10 (27.0)	0.51
Without invasive examination	30 (8.0)	18 (60)	12 (40)	0.32
Others	5 (1.3)	3 (60)	2 (40)	0.69
Reasons for non-CAM use (n = 169)				
Terrible taste	59 (34.9)	12 (20.3)	47 (79.7)	0.14
Slower effectual procedure	77 (45.6)	9 (11.7)	68 (88.3)	0.30
Afraid of fake CAM products	29 (17.2)	4 (13.8)	25 (86.2)	0.87
Doubt of effect	104 (61.5)	2 (1.9)	102 (98.1)	<0.01
Inconvenient	94 (55.6)	15 (16.0)	79 (84.0)	0.63
Afraid of acupuncture	23 (13.6)	4 (17.4)	19 (82.6)	0.71
Others	7 (4.1)	2 (28.6)	5 (71.4)	0.29
CAM preferred for future (n = 169)				
Patent herbal medicine	137 (81.1)	23 (16.8)	114 (83.2)	0.13
Herbal decoction	54 (32.0)	12 (22.2)	42 (77.8)	0.06
Nutrient supplements	112 (66.3)	18 (16.1)	94 (83.9)	0.51
Exercise	36 (21.3)	8 (22.2)	28 (77.8)	0.15
Acupuncture	40 (23.7)	9 (22.5)	31 (77.5)	0.12
Others	13 (7.7)	4 (30.8)	9 (69.2)	0.09

CM, Chinese medical; WM, western medical; CAM, complementary and alternative medicine; OR, odds ratio; Cl, confidence interval.

### Differences of CAM use features between CM and WM hospitals

Among these 377 patients who used CAM in the management of their CAD, 257 (68.2%) were from CM hospitals and 120 (31.8%) were from WM hospitals. As expected, the proportion of CAM users in patients from CM hospitals was significantly higher than that in WM hospitals (91.1% vs. 45.5%, *P* < 0.01). In addition, significant differences were also found between CM and WM hospital CAM users in terms of CAM use frequency (*P* < 0.01), types (herbal decoction: *P* < 0.01; acupuncture: *P* = 0.04), perceived effectiveness (*P* < 0.01), as well as reasons for CAM use (effective: *P* < 0.01; treating the root cause: *P* < 0.01) and non-CAM use (doubt of effect: P < 0.01). CAM users from CM hospitals were more likely to be continuous users (p < 0.01; OR = 4.17, 95% CI = 2.41 - 7.22), use herbal decoction (p < 0.01) and acupuncture (p = 0.04), believe in the efficacy of CAM treatments (p < 0.01; OR = 4.01, 95% CI = 2.45 - 6.58) [see Table 3].

## Discussion

This study provides one of the first comprehensive investigations of CAM use in the Chinese population who were diagnosed with CAD. The present study confirmed the hypothesis that more patients in CM hospitals use CAM than WM hospitals. The prevalence of CAM use among Chinese patients with CAD was 69% in the present study. It is higher than 4% - 61% in patients with cadiovascular disease as shown by Grant et al. [20]. Patent herbal medicine was the most commonly used CAM type (90.7% of all CAM users) in the present study; whereas Grant et al. [20] found that herbal medicines were used by only 2% to 46% patients with cardiac diseases. Upon a close examination, the data in the meta-analysis by Grant et al. [20] were mainly based on studies performed in the United States, Canada, and the United Kingdom. Thus, the cause of differences may be due to different cultural backgrounds. The results of the present study indicate that physicians should be aware of the high prevalence of CAM use, most likely herbal medicine use, in the Chinese populations.

The present study indicates that old male patients with UA are more likely to be CAM users and CAM users are less likely to be hospitalized or experience emergency admission in the past one year. These results may be influenced by age, gender and/or disease duration. Nonetheless, as CAM users were found to be older, have a longer disease duration, and better current health status than non-CAM users, we may hypothesize that CAM may alleviate patients’ symptoms of CAD. This was confirmed with patients’ perceived effectiveness of CAM in the present study. However, evaluation of CAM effectiveness in the management of CAD is beyond the purposes of the present study and further high quality randomized control trials are needed to explore the possible therapeutic effectiveness of CAM especially herbal medicine for CAD.

In the present study, CAM users were older; whereas gender and education were not associated with CAM use. Interestingly, in western populations, CAM users were found to be more likely young, female, and with higher education [11,13,15-19]. The variances may be related to the cultural background. During the past thirty years, China has undergone tremendous westernization. The young generation, as compared to the old generation, has been exposed more to western culture including strong believes in western medicine and lack of connection with traditional Chinese medicine. As traditional Chinese medicine is the major type of CAM in China and the sole type of medicine in some Chinese rural areas, the old generation thus may tend to believe and use CAM more than the young generation.

The present study demonstrated that a variety of CAM therapies, including patent herbal medicine, herbal decoction, acupuncture, and exercise, are prevalent and widely used in patients with CAD from both CM and WM hospitals in Beijing. Nonetheless, the small portion of other CAM types indicates that unlike the results provided by Prasad et al. [19], chiropractor, mind-body treatment, and massage are not commonly used by the Chinese patients who were diagnosed with CAD. The reasons may be: 1) chiropractic therapy is not common in China and/or 2) Chinese patients don’t believe that these forms of CAM work for CAD. Regarding the frequency of use of specific types of CAM, the results of the present study differ from the results reported by Prasad et al. [19]. In the present study, patent herbal medicine and herbal decoctions are the two most commonly used CAM therapies; whereas dietary supplements and chiropractic therapy are the two most commonly used in the study by Prasad et al. [19]. The difference may be due to the strong cultural influence. Compared with other types of CAM therapies, the Chinese population including the Chinese physicians and Chinese patients may believe that herbal medicine is more effective for the management of CAD.

Decker et al. [18] reported that higher portions of patients with cardiovascular diseases were uninsured and economically burdened. The present study found opposite results as the majority of the patients were urban citizens and the majority of patients reported a monthly household income higher than 4,000 RMB. The possible reason for the results may be due to the location of the sampled hospitals which locate in urban Beijing. In order to test the difference of economic statuses of CAM use populations, hospitals locate in the suburb or countryside should be included. The present study also found that the majority of CAM users tend to use CAM intermittently or once. As the majority of CAM users in the present study had higher monthly house hold income, causes of intermittent use may be due to cultural believes (Chinese people may believe intermittent use may be more effective) or physician instructions or just compliance in addition to economic costs. The main cause of CAM use in the present study was patients’ belief of few side effects whereas the main cause of non-CAM use was doubt of effects. Nonetheless, modern research studies report that CAM therapies like herbal medicine does have side effects [26], and certain CAM therapy have been shown to be effective in the management of certain disorders, such as acupuncture for pain [27]. Consequently, in order to correct the misunderstandings of CAM of the general public, physicians should give the Chinese patients a more detailed introduction of CAM therapy from an evidence-based approach upon clinical treatment of CAD.

### Limitations

Although the study included 377 CAM users which may be sufficient for the analysis of features of CAM use in patients with CAD in Beijing, patients were collected only in the cardiac outpatient clinics of four tertiary-level hospitals out of 35 tertiary-level hospitals. Patients with CAD relying solely on other CAM therapies besides traditional Chinese medicine may not be included in the present study. Therefore, the features captured in the present study may not well characterize the features of CAM use in patients diagnosed with CAD in Beijing. Patients were asked to complete a questionnaire through recalling the use of CAM in the past years; recall bias may exist during the data collection process. In addition, the validity and reliability of Part One of the questionnaire used in the study require further examination.

## Conclusions

The present study suggested a group of significant factors which could influence the use of CAM in Chinese patients with CAD. CAM use patterns differ in patients from CM and WM hospitals. The study may help clinicians understand the preferred options and perspectives of CAD treatments by the Chinese patients.

## Competing interests

All authors declare that they have no competing interests.

## Authors’ contributions

HXL and JW designed and coordinated the study. FYC, XY, ZZ, and XJX carried out the survey study. XJX performed the statistical analysis. FYC and XY drafted the manuscript. All authors read and approved the final manuscript.

## Pre-publication history

The pre-publication history for this paper can be accessed here:

http://www.biomedcentral.com/1472-6882/13/287/prepub
